# Dataset of microscale atmospheric flow and pollutant concentration large-eddy simulations for varying mesoscale meteorological forcing in an idealized urban environment

**DOI:** 10.1016/j.dib.2025.111285

**Published:** 2025-01-10

**Authors:** Eliott Lumet, Thomas Jaravel, Mélanie C. Rochoux

**Affiliations:** CECI, Université de Toulouse, CNRS, CERFACS, 42 Avenue Gaspard Coriolis cedex 1, 31057 Toulouse, France

**Keywords:** Microscale meteorology, Urban flow, Atmospheric dispersion, Meteorological uncertainty, Internal variability, Large-eddy simulation, Perturbed-parameter ensemble

## Abstract

By 2050, two-thirds of the world's population will live in urban areas under climate change, exacerbating the environmental and public health risks associated with poor air quality and urban heat island effects. Assessing these risks requires the development of microscale meteorological models that quickly and accurately predict wind velocity and pollutant concentration with high resolution, as the heterogeneity of urban environments leads to complex wind patterns and strong pollutant concentration gradients. Computational Fluid Dynamics (CFD) has emerged as a powerful tool to address this challenge by providing obstacle-resolved flow and dispersion predictions. However, CFD models are very expensive and require intensive computing resources, which can hinder their systematic use in practical engineering applications. They are also subject to significant uncertainties, particularly those arising from the mesoscale meteorological forcing and the internal variability of the atmospheric boundary layer, some of which are aleatory and thereby irreducible. Given these issues, the construction of CFD datasets that account for uncertainty would be an interesting avenue of research for microscale atmospheric science.

In this context, we present the PPMLES (Perturbed-Parameter ensemble of MUST Large-Eddy Simulations) dataset, which consists of 200 large-eddy simulations (LES) characterizing the complex interactions between the turbulent airflow, the tracer dispersion, and an idealized urban environment. These simulations reproduce the canonical MUST dispersion field campaign while perturbing the model's mesoscale meteorological forcing parameters. PPMLES includes time series at human height within the built environment to track wind velocity and pollutant release and dispersion over time. PPMLES also includes complete 3-D fields of first- and second-order temporal statistics of the wind velocity and pollutant concentration, with a sub-metric resolution. The uncertainty of the fields induced by the internal variability of the atmospheric boundary layer is also provided. The computation of PPMLES required significant resources, consuming 6 million CPU core hours, equivalent to the emission of approximately 10 tCO2eq of greenhouse gases. This significant computational effort and associated carbon footprint motivates the sharing of the data generated.

The added value of the PPMLES dataset is twofold. First, the perturbed-parameter ensemble of LES enables to quantify and understand the effects of the mesoscale meteorological forcing and the internal variability of the atmospheric boundary layer, which has been identified as a major challenge in predicting atmospheric flow and pollutant dispersion in urban environments. Secondly, PPMLES reference data can be used to benchmark models of different levels of complexity, and to extract key information about the physical processes involved to inform more operational modeling approaches, for example through learning surrogate models.

Specifications Table

**Table:** 

Subject	Atmospheric Science
Specific subject area	Large-eddy simulations of microscale wind flow and pollutant concentration in an idealized urban environment and for varying mesoscale meteorological forcing
Type of data	Dataset (HDF5), Table (CSV), Raw and processed simulation results.
Data collection	The data were obtained by running an ensemble of 200 large-eddy simulations reproducing the MUST field trial #2681829 thanks to the AVBP# solver. The simulations were run on four different supercomputers: CERFACS’ Nemo (Intel Haswell) and Kraken (Intel Skylake), Météo-France's Belenos (AMD Rome), and TTGC's Joliot-Curie (Intel Skylake/AMD Rome). No simulation was excluded, and the raw results were post-processed to provide temporal statistics and uncertainty estimates.
Data source location	CECI, Université de Toulouse, CNRS, CERFACS
Data accessibility	Repository name: PPMLES – Perturbed-Parameter ensemble of MUST Large-Eddy Simulations Data identification number: 10.5281/zenodo.11394347 Direct URL to data: https://zenodo.org/records/11394347
Related research article	None*.*

# AVBP LES code [7],https://www.cerfacs.fr/avbp7x/ (Accessed 2025-01-02).

## 1 Value of the Data

• These data are useful for understanding the complex interactions between the atmospheric boundary layer and the dispersion of pollutants in urban environments, through the example of the canonical MUST field experiment, which corresponds to an idealized urban environment made of regularly-spaced shipping containers.
• The dataset consists of a perturbed-parameter ensemble of 200 high-fidelity large-eddy simulations, with each simulation sample corresponding to a different mesoscale meteorological forcing to provide an indication of the envelope of possible microscale urban flow and pollutant concentration scenarios.
• The dataset includes time series at human height to track the pollutant release and dispersion over time as well as complete 3-D fields of time-averaged statistics of the steady-state wind velocity and pollutant concentration at a high spatial resolution (sub-meter), together with the associated uncertainties.
• These data can be used as learning data to train surrogate models, allowing researchers to experiment with new machine learning architectures to accelerate the prediction of microscale atmospheric processes.
• Researchers can use and potentially extend this dataset for multi-model comparisons to assess the structural uncertainty in large-eddy simulations.

## 2 Background

The PPMLES (Perturbed-Parameter ensemble of MUST Large-Eddy Simulations) dataset was originally computed in [1] to better understand the near-field dispersion of air pollutants in an idealized urban environment and at a very high resolution (i.e., sub-meter scale), and to gain insight into its sensitivity to mesoscale meteorological forcing.

Although several datasets of wind tunnel measurements of pollutant concentrations in idealized urban environments are available (e.g. CEDVAL^1^), they cannot represent the full range of atmosphere-urban interactions. Field-scale experiments are more representative but they are costly, their mesoscale conditions cannot be controlled, and they provide data that are spatially scarce. This has motivated the construction of an LES dataset with high spatio-temporal resolution and for a wide range of mesoscale meteorological forcing. The selected case, the MUST campaign [2,3], has been used for a multi-model intercomparison [4], but access to the simulation data has not been maintained.

The PPMLES dataset was used to train a surrogate model that emulates the response surface of the LES model [1,5]. This surrogate, which makes instantaneous predictions, was then used in a data assimilation framework to reduce the uncertainty in pollutant concentration predictions using local measurements.

## 3 Data Description

The PPMLES dataset is a perturbed-parameter ensemble of 200 large-eddy simulations (LES) of wind flow and pollutant dispersion in the canonical MUST idealized urban environment corresponding to an array of regularly-spaced shipping containers [2,3]. Each LES replicates the MUST field experiment for a different mesoscale meteorological forcing, which is parameterized with two uncertain input parameters: i) the inlet wind direction $\alpha_{inlet}$, which is assumed uniform and homogeneous, and ii) the friction velocity $u_{*}$, which scales the logarithmic inlet wind profile representing a fully developed neutral atmospheric surface layer.

**Fig. 1:**
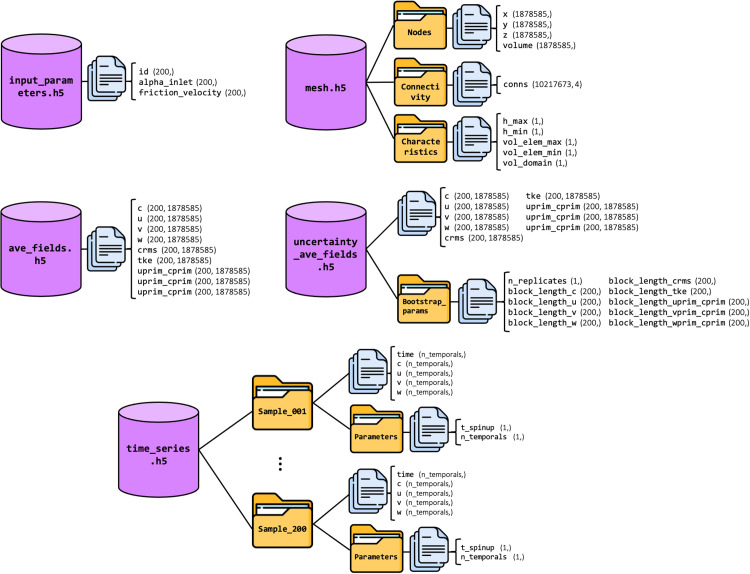
Organization of HDF5 files, represented as purple cylinders and listed in Table 1. Each file consists of groups and/or datasets represented by yellow folders and blue files. The name of each dataset is shown along with its shape in parentheses.

An overview of the dataset files is given in Table 1. Except for the probe network definition (in CSV), all data is stored in HDF5^2^ files. This format provides efficient storage, fast access, and hierarchical data organization. Fig. 1 gives a comprehensive description of the structure of the HDF5 files in PPMLES.

**Table 1:** General description, size and type of each file in the dataset.

Filename	Description	Size	Type
input_parameters.h5	List the 200 meteorological forcing input parameters (wind direction and friction velocity).	6.8 ko	HDF5
ave_fields.h5	List of the main time-averaged wind velocity and tracer concentration fields predicted for each input parameter sample.	17.1 Go	HDF5
uncertainty_ave_fields.h5	List of the uncertainty of each time-averaged field as standard deviation and for each input parameter sample.	15.9 Go	HDF5
mesh.h5	Contains the definition of the mesh on which the fields are discretized.	387 Mo	HDF5
time_series.h5	List of the main wind and tracer concentration time series predicted by LES for each input parameter sample at 93 probe locations.	3.1 Go	HDF5
probe_network.csv	Contains the coordinates of each probe on which time series are saved.	2.9 ko	CSV

**Fig. 2:**
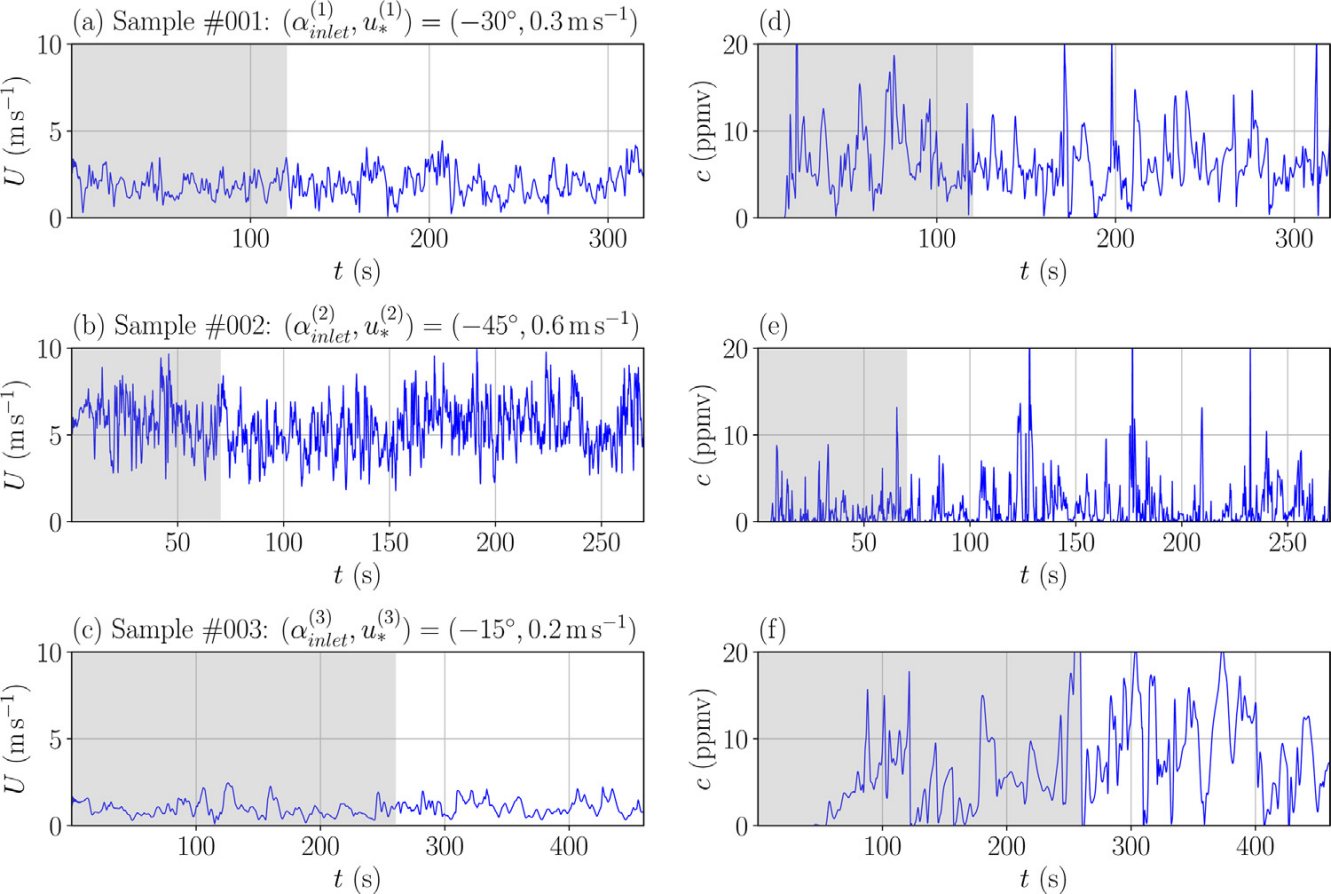
Time series of the wind speed magnitude U (a, b, c) and propylene concentration c (d, e, f) predicted by LES at tower B at *z* = 2 m within the array of containers (see Fig. 5b). Results are shown for the first three samples of the perturbed-parameter ensemble. Shaded gray areas correspond to the spin-up time used for each simulation.

For each sample, the predicted time-series of tracer concentration *c* (in ppmv) and wind velocity components *u, v, w* (in m.s^-1^) at 93 probe locations within the array of containers are stored in time_series.h5. The probe locations are defined in probe_network.csv. The coordinate system used is the same as in [3], so that the *x*-*y* axis system is aligned with the containers array. Examples of the wind velocity magnitude and tracer concentration time series for three different samples are shown in Fig. 2. Note that the simulation spin-up time is included in each time series and is adapted to the friction velocity, which implies that the time series duration (n_temporals) is different for each sample.

The time-averaged fields of wind velocity and tracer concentration statistics are reported in the file ave_fields.h5. These 3-D fields are discretized over a mesh of $N_{nodes}$ = 1878,585 nodes, with a resolution of 30 cm between the containers, allowing to have at least 8 cells over the height of each container. The coordinates and dual volume of each node are reported in the file mesh.h5. The 3-D fields are given as arrays of dimensions ($N_{samples}$, $N_{nodes}$), where $N_{samples}$ = 200 is the number of LES simulations. The dataset includes the fields of the following statistics of interest:

i. First-order statistics: the time-averaged tracer concentration *c* (ppmv) and wind velocity components *u, v, w* (m.s-1),
ii. Second-order statistics:
  ○ The concentration root mean square fluctuations crms = $\sqrt{\bar{c^{{}^{\prime }2}}}=\sqrt{\bar{{\left(c-\bar{c}\right)}^{2}}}$ (ppmv), where the upper bar denotes time-averaged quantities,
  ○ The turbulent kinetic energy of the wind tke = $\frac{1}{2}\left(\bar{u^{{}^{\prime }2}}+\bar{v^{{}^{\prime }2}}+\bar{w^{{}^{\prime }2}}\right)$ (m2.s-2),
  ○ The tracer turbulent transport components uprim_cprim, vprim_cprim, and wprim_cprim (ppm.m.s-1), defined as $\bar{u^{\prime }c^{\prime }}$, $\bar{u^{\prime }c^{\prime }}$, and $\bar{w^{\prime }c^{\prime }}$.

**Fig. 3:**
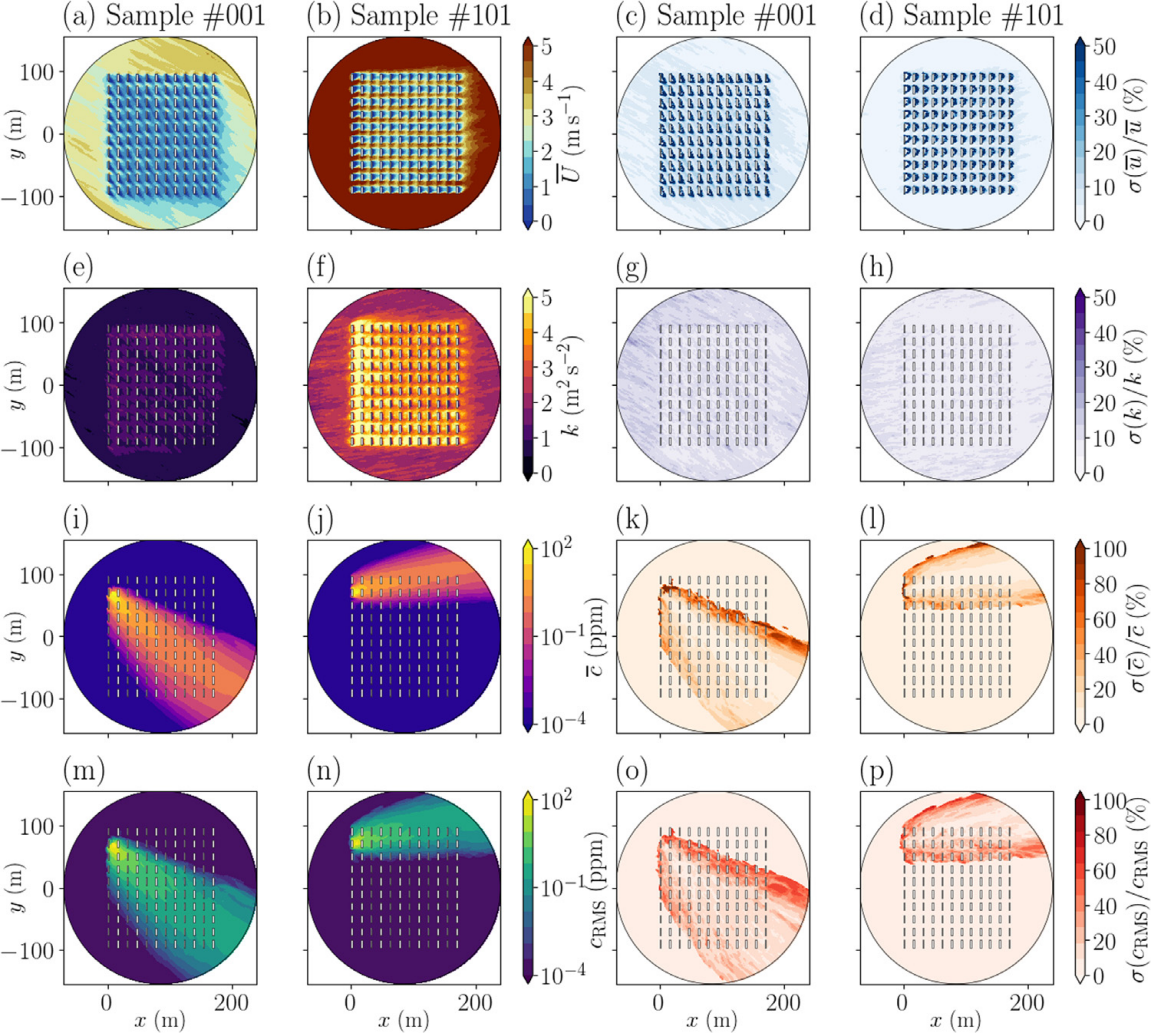
Horizontal cuts at *z* = 1.6 m of the time-averaged wind speed magnitude $\bar{U}$ (a, b), turbulent kinetic energy k (e, f), concentration $\bar{c}$ (i, j) and concentration fluctuation $c_{\mathrm{RMS}}$ (m, n) fields, along with their associated uncertainty as relative standard deviation (c, d, g, h, k, l, o, p). Results are shown for the sample #001 with $\left(\alpha_{\mathrm{inlet}}^{\left(1\right)},u_{*}^{\left(1\right)}\right)=\left(-30\text{°},\;0.3\;m/s\right)$ on the first and third columns (corresponding to the time series in Fig. 2a,d), and for the sample #101 $\left(\alpha_{\mathrm{inlet}}^{\left(101\right)},u_{*}^{\left(101\right)}\right)=\left(9\text{°},\;0.7\;m/s\right)$ on the second and last columns. White rectangles represent containers from the MUST field campaign.

**Fig. 4:**
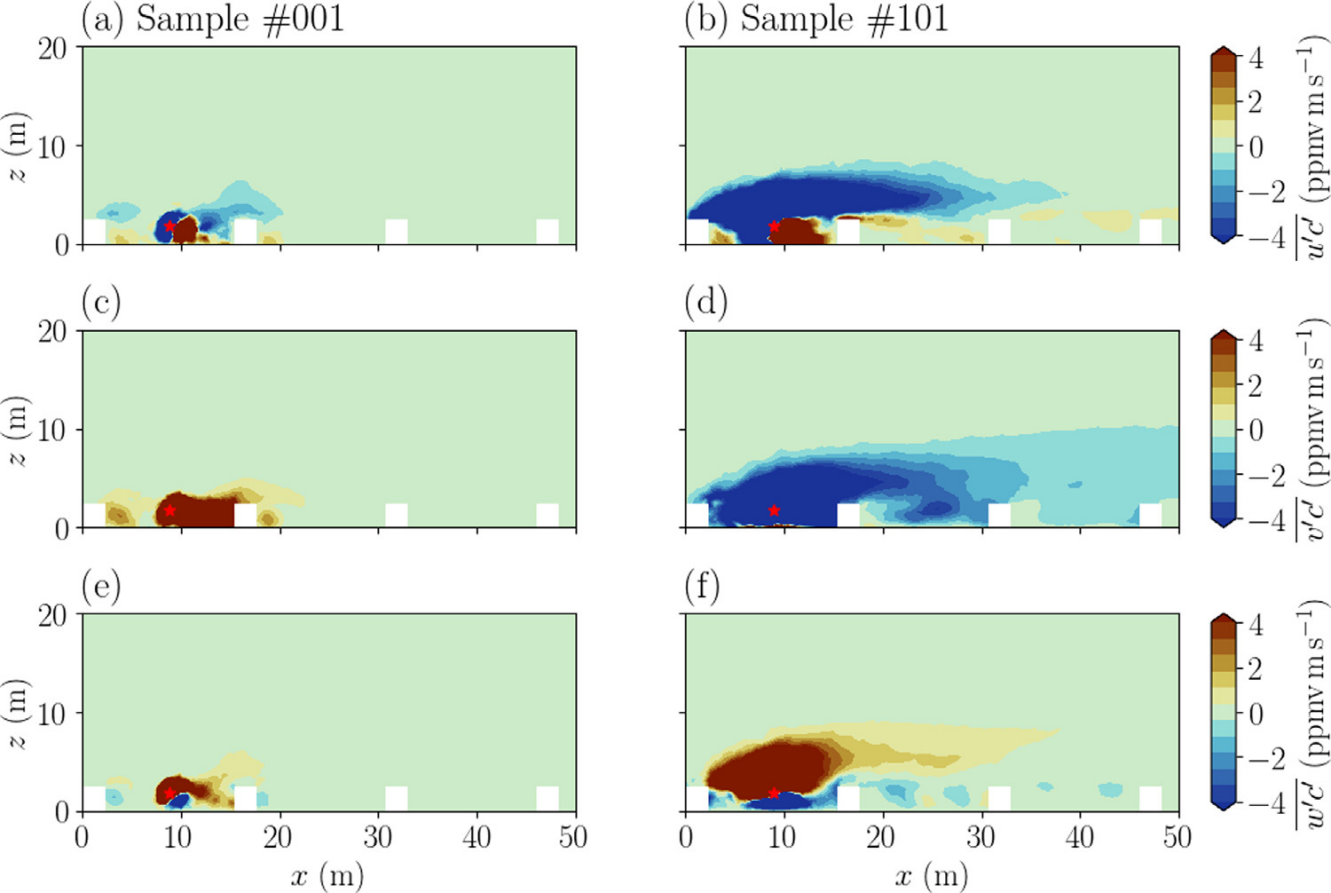
Vertical cuts crossing the tracer source location (represented by the red star) of the time-averaged turbulent tracer transport components $\bar{u^{\prime }c^{\prime }}$ (a, b), $\bar{v^{\prime }c^{\prime }}$ (c, d), $\bar{w^{\prime }c^{\prime }}$ (e, f) fields. Results are shown for the sample #001 with $\left(\alpha_{\mathrm{inlet}}^{\left(1\right)},u_{*}^{\left(1\right)}\right)=\left(-30\text{°},\;0.3\;m/s\right)$ on the left column (corresponding to the time series in Fig. 2a, d and to the horizontal fields in Fig. 3, columns 1 and 3), and for the sample #101 $\left(\alpha_{\mathrm{inlet}}^{\left(101\right)},u_{*}^{\left(101\right)}\right)=\left(9\text{°},\;0.7\;m/s\right)$ on the right column (corresponding to the horizontal fields in Fig. 3, columns 2 and 4). White rectangles represent containers from the MUST field campaign.

Time averages are collected over a 200-s analysis period, which is the standard duration for the MUST case study [3,4]. Examples of these statistic fields are given as horizontal cuts in Fig. 3 (columns 1 and 2) and vertical cuts in Fig. 4 for two samples of the ensemble.

Calculating the temporal statistics over a limited analysis period (i.e. 200 s in the MUST experiment) introduces a significant uncertainty due to the internal variability of the atmospheric boundary layer [4,6],. The file uncertainty_ave_fields.h5 provides an estimate of this aleatory uncertainty for each field in ave_fields.h5, as a standard deviation field discretized over the same 3-D mesh as the physical fields. The uncertainty is estimated during the simulation post-processing using a bootstrap approach (Section 4.1). The bootstrap parameters, i.e. the number of replicates n_replicates and the length of the block_length used for each field, are specified in the Bootstrap_params group of the uncertainty_ave_fields.h5 file. Examples of the aleatory uncertainty, as relative standard deviation, are given in Fig. 3 (columns 3 and 4) for two samples of the ensemble.

## 4 Experimental Design, Materials and Methods

In this section, we provide a comprehensive description of the design and methods used to acquire the PPMLES dataset. We first introduce the case study and the large-eddy simulation (LES) model used to generate the PPMLES dataset. We then explain the design of the perturbed-parameter ensemble and how the model was modified to simulate the ensemble of wind and pollutant dispersion scenarios. Finally, we retrace all the post-processing applied to the raw simulation results to obtain the data available in the PPMLES dataset and we give an estimate of its carbon footprint.

### 4.1 Large-eddy simulation model of the MUST field trial 2681829

#### 4.1.1 The MUST field campaign

**Fig. 5:**
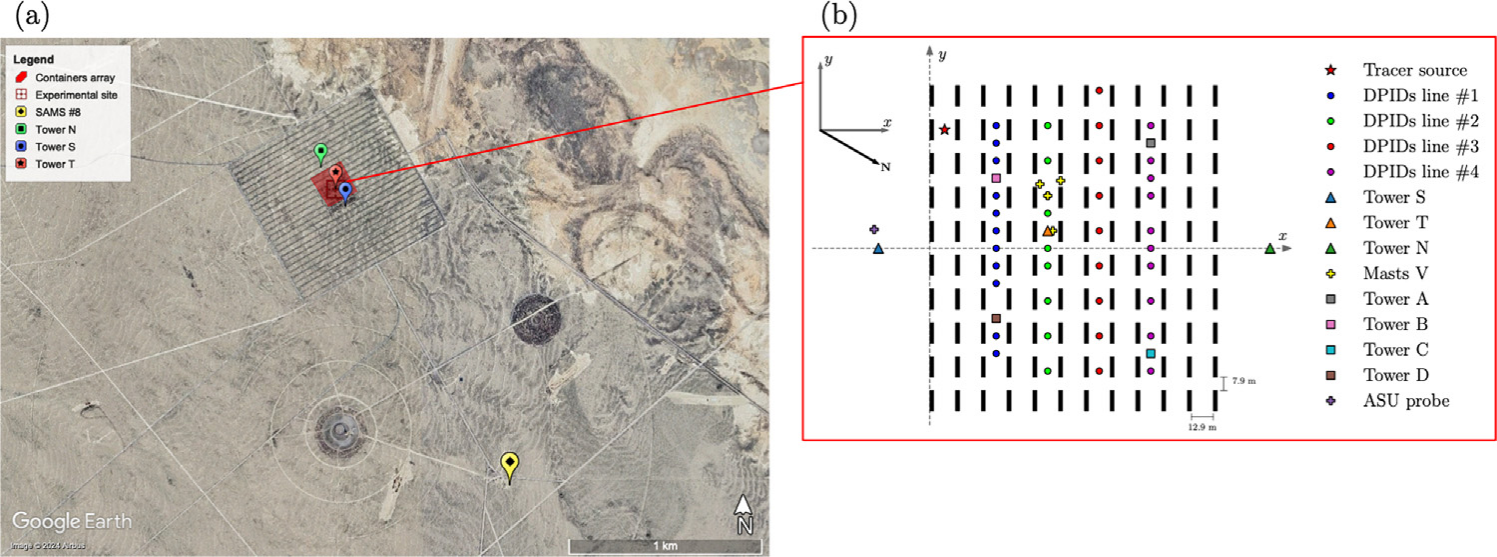
(a) Satellite image of the Dugway Proving Ground test site where the MUST field campaign was conducted. The red crosshair indicates the location of the simplified urban canopy area made of containers. The location of the SAMS meteorological #8 and towers S, T, N are indicated by the yellow, blue, red and green markers, respectively. (b) Close-up schematic view showing the location of each sensor for which time series are stored. The location of the propylene emission source for the MUST trial 2681829 is shown as a red star.

MUST is a field experimental campaign conducted in September 2001 at the US Army Dugway Proving Ground test site in the Utah desert, USA (Fig. 5a). Its goal was to collect comprehensive measurements within an idealized urban canopy to support the development and validation of urban dispersion models [2,3]. During the experiments, a non-reactive gas tracer (propylene) was released within an idealized urban canopy consisting of an array of 10 × 12 regularly-spaced shipping containers covering an area of approximately 200 × 200 m^2^ (Fig. 5b). The containers are 12.2-m long, 2.42-m wide, and 2.54-m high. Fig. 5 shows the location of the towers and masts carrying the wind velocity and tracer concentration sensors used during the campaign. For a full description of the instruments used, the reader is referred to [2].

MUST is a canonical field-scale dispersion experiment that has been used to validate a large number of CFD dispersion models and for the COST Action 732 CFD model intercomparison [4]. For the construction of the PPMLES dataset, we focus on the trial 2681829, which corresponds to neutral atmospheric conditions and to the tracer source location shown in Fig. 5b.

#### 4.1.2 The microscale obstacle-resolving flow modeling approach

To build the PPMLES dataset, we use as a reference the LES model of the MUST trial 2681829 validated in [6]. This model uses AVBP^3^ to solve the LES-filtered Navier-Stokes and tracer advection-diffusion equations, with a second-order Lax-Wendroff finite-volume centered numerical scheme [7]. Subgrid-scale turbulence is modeled using the Wall-Adaptative Local Eddy-Viscosity model [8] for subgrid momentum transport, and a gradient-diffusion hypothesis for subgrid tracer transport (with a turbulent Schmidt number of 0.6). We also use a pressure gradient scaling (PGS) for low Mach number flows to reduce computational cost [9].

**The computational domain** of the reference LES model is a rectangular parallelepiped oriented along the mean streamwise wind direction, with dimensions of 420 × 420 × 50 m^3^ (represented by the red square in Fig. 8). The domain was discretized using the CENTAUR^4^ mesh generator resulting in an unstructured and boundary-fitted mesh of 91 million tetrahedra. In the region of interest, which corresponds to a 246 × 266 × 3.6 m^3^ box containing all the containers, the mesh is uniform with a resolution of 0.3 m. This resolution ensures that there are 8 cells over the height of each container, a minimal requirement to accurately predict their effect on the flow. In the rest of the domain, the mesh is gradually stretched to reach a resolution of 5 m at the top boundary, with a maximum stretching ratio of 1.7, to further save computational time.

**Boundary conditions**. At the inlet, a logarithmic vertical wind profile is imposed so that the mean inlet wind velocity vector $\bar{u_{inlet}}$ reads

(1) $$\bar{u_{inlet}}=\left(\begin{array}{c} \bar{u_{inlet}}\cos\left(\alpha_{inlet}\right)\\ \bar{u_{inlet}}\sin\left(\alpha_{inlet}\right)\\ 0 \end{array}\right),\;with\;\bar{u_{inlet}}\left(z\right)=\frac{u_{*}}{\kappa }\ln\left(\frac{z+z_{0}}{z_{0}}\right),$$

where $u_{*}$ is the friction velocity, κ is the von Kármán constant equal to 0.4, and $z_{0}$ is the aerodynamic roughness length, which was estimated to be 0.045±0.005 m for the MUST field terrain [3]. In addition, a synthetic turbulence injection method [10] is used to impose upstream wind fluctuations, which are calibrated using a precursor simulation (with periodic boundary conditions and no obstacles) [6]. Free slip boundary conditions are used at the lateral boundaries. Static pressure is imposed at the outlet and top boundaries. A smooth law of the wall is used to impose the shear stress at the obstacle boundaries, while at the ground boundaries the shear stress is imposed according to the Monin-Obhukov similarity theory in neutral conditions to match the experimentally estimated aerodynamic roughness length $z_{0}$. The tracer source is modeled by a source term in the advection-diffusion equation that matches the experimental volumetric flow rate. A full description of the boundary conditions is given in [1].

**Initial conditions**. The LES simulation is initialized with a homogeneous flow field in the horizontal direction equal to the prescribed inlet mean field (Eq. (1)). To ensure that first- and second-order statistics of the flow and the tracer reach a stationary state, we use a spin-up of 1.5 times the convective time scale, which is about 17 times the LES turnover time $H/u_{*}$ with *H* the height of the containers, before collecting the statistics.

**The reference simulation** is defined by setting the mesoscale meteorological forcing parameters thanks to the field campaign upstream wind velocity measurements at tower S and ASU probe (Fig. 5b). It yields $\alpha_{inlet}^{\left(ref\right)}$ = −41° and $u_{*}^{\left(ref\right)}$ = 0.73 m.s^-1^. Concerning the temporal resolution, the time step imposed by the numerical scheme is equal to 7.9 × 10^–4^ s when using PGS. At the probe locations (Fig. 5b), the outputs are stored with a resolution of 0.05 s. For the full 3-D fields, instantaneous fields were not saved to limit the amount of data stored (apart from those needed to restart simulations), and sliding time-averaged fields over a 10-s period are saved for uncertainty estimation. Note that thresholding is not applied to physical quantities that may be negative due to numerical errors, such as the tracer concentration, to ensure conservation. The final LES predictions of wind velocity and tracer concentration statistics are defined over a 200-s analysis window as in [3,4], so that they can be compared with field measurements. Note that this limited acquisition time introduces a significant aleatory uncertainty in the LES predictions (Section 4.1).

### 4.2 Perturbed-parameter ensemble design

#### 4.2.1 Definition of the input parameter space

To explore the sensitivity of the wind velocity and pollutant concentration statistics to the mesoscale meteorological forcing, we design an ensemble of LES by perturbing the boundary condition parameters that have the most influence on the predictions under neutral thermal stratification conditions [1]: the inlet wind direction $\alpha_{inlet}$ and the friction velocity $u_{*}$. These parameters determine the vertical profile imposed at the inlet boundary condition (Eq. (1)). Note that the level of turbulence imposed at the inlet has a negligible effect on the LES predictions as the turbulence spectrum quickly returns to an equilibrium state with the rough ground [1].

**Fig. 6:**
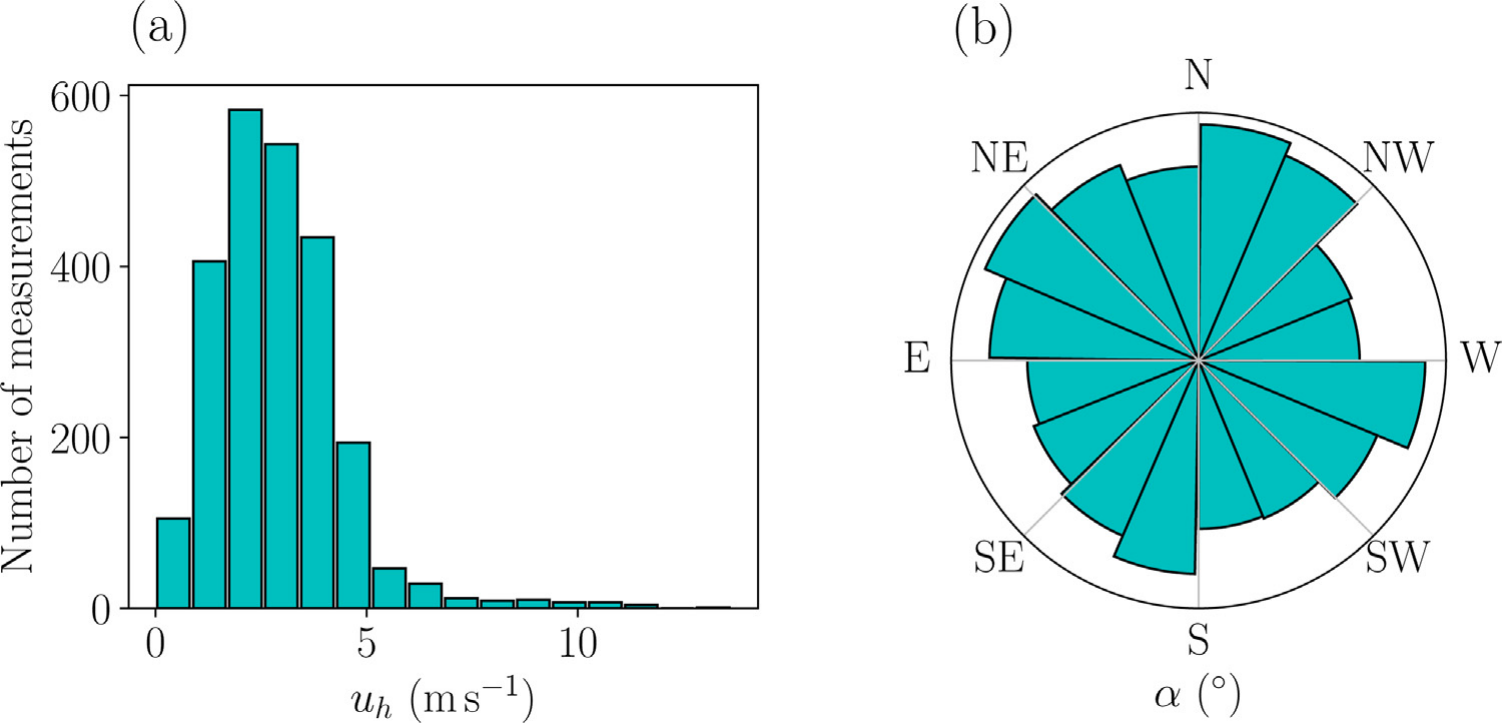
Distributions of the horizontal wind velocity (a) and wind direction (b) based on 15-minute averaged wind measurements at the SAMS meteorological station #8 (Fig. 5a) at *z* = 10 m over 12 days during the MUST field campaign.

We then define a plausible range of variation for these two input parameters $\left(\alpha_{inlet},u_{*}\right)$ thanks to a microclimatology using available measurement data from the nearest meteorological station, i.e. the SAMS station #8 located approximately one kilometer from the MUST field campaign site (Fig. 5a). This represents a total of 2391 15-minute averaged wind measurements at 10 m above ground level. Fig. 6 shows that all wind directions are likely to occur and that >99 % of the horizontal wind speed measurements are below 12 m.s^-1^, corresponding to a friction velocity of 0.89 m.s^-1^. For the ensemble generation, we thereby limit the maximum friction velocity to 0.89 m.s^-1^ and we also limit the minimum friction velocity to 0.07 m.s^-1^, which corresponds to a wind speed of about 1 m.s^-1^ at 10 m height to focus on windy conditions. The range of variation for the inlet wind direction $\alpha_{inlet}$ is also narrowed so that the pollutant plume always remains mostly in the canopy and therefore at the level of existing sensors in the LES simulations. The input parameter space thus reads:

(2) $$\left(\alpha_{inlet},u_{*}\right)\in \Omega =\left[-90^{\circ },\;30^{\circ }\right]\times \left[0.07\;m\;s^{-1},0.89\;m\;s^{-1}\right].$$

#### 4.2.2 Sampling of the input parameter space

**Fig. 7:**
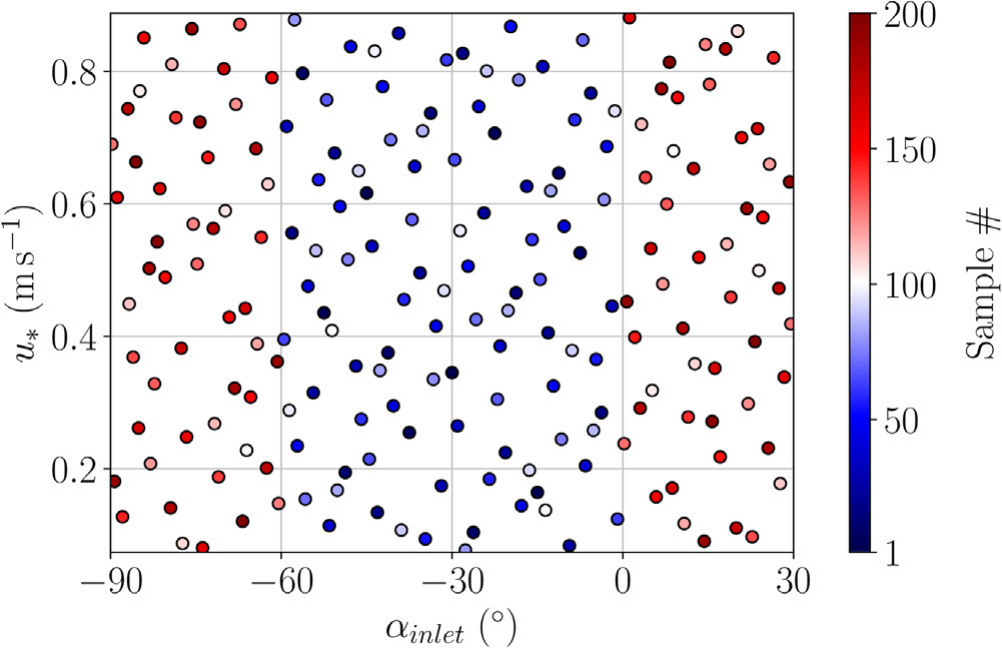
Input parameter space sampling obtained with Halton's sequence. Each point is a pair of parameters for which we perform an LES prediction.

The next step is to sample the input parameter space Ω and run one LES per sample to generate the PPMLES dataset. Given the very large computational cost of the LES model, our computational budget was 200 simulations. To get the most out of this budget, we use the Halton's sequence [11] to sample the input parameter space as uniformly as possible. Indeed, as a low-discrepancy sequence, it covers the input parameter space more efficiently than a purely random sequence by avoiding sampling the same region multiple times. For practical reasons, the input parameter ensemble was generated in two parts: the first 100 samples corresponding to angles between −60° and 0°, and the next 100 samples corresponding to angles in $\left[-90^{\circ },-60^{\circ }\left[\cup \right]0^{\circ },30^{\circ }\right]$. Fig. 7 shows the resulting perturbed-parameter ensemble colored by sample index.

### 4.3 Model adaptation for perturbed-parameter ensemble generation

In this section, we detail the modifications made in the LES model to predict the fields of interest for every input parameter sample in the perturbed-parameter ensemble (Fig. 7).

#### 4.3.1 Computational domain adaptation to the wind direction

**Fig. 8:**
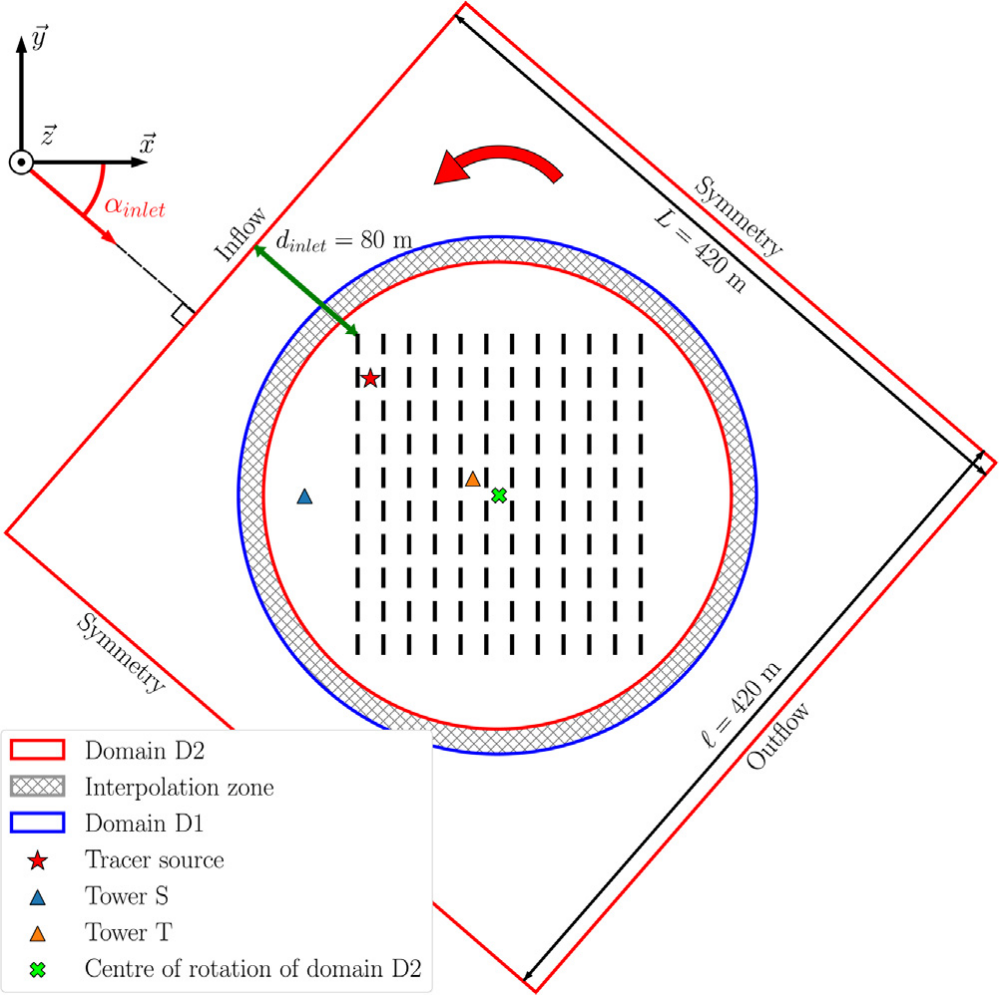
Horizontal schematic view of the computational domain at the level of the containers. The domain is divided into two subdomains: the fixed domain D1 in blue and the rotating peripheral domain D2 in red. The interpolation overlap area between D1 and D2 used for the coupling with the CWIPI library is hatched. The coordinate system shown is the one defined by [3] and is attached to the fixed domain D1. The location of the tracer source in the MUST trial 2681829 is indicated by the red star. The blue (resp. orange) triangle symbol represents the tower S (resp. T).

In the reference LES model, if the mean flow direction deviates too much from the reference wind direction value, it induces lateral confinement and numerical instabilities due to the free slip boundary conditions at the domain sides. This problem is solved by rotating the computational domain so that the sides always remain parallel to the mean flow direction. To efficiently implement this feature, the domain is split into two subdomains as shown in Fig. 8: the peripheral domain D2, which is rotated to align with $\alpha_{inlet}$, and the inner domain D1, which is fixed.

The Navier–Stokes equations are solved on each domain by parallel AVBP instances [12], coupled using CWIPI.^5^ The interpolation between the two domains is computed over an overlapping region (hatched area in Fig. 8). This region should contain at least 10 cells between the subdomain boundaries in each direction, resulting in a 13 % increase in the number of cells in the computational mesh.

This domain decomposition facilitates the generation of a large ensemble of simulations because it does not require the generation of a new mesh for each new wind direction. In addition, having a static internal domain shared by all LES simulations avoids the use of interpolation to compare LES predictions obtained with different wind conditions.

#### 4.3.2 Turbulence injection rescaling

In the reference LES model, a precursor simulation without obstacles is performed to estimate the Reynolds stress tensor and inject realistic wind fluctuations at the inlet [6]. To avoid running a precursor simulation for each pair of input parameters $\left(\alpha_{inlet},u_{*}\right)$ in the Halton's sequence, the parameter-dependent Reynolds stress tensor $R(\alpha_{inlet},u_{*})$ is obtained by rotating and then rescaling the reference Reynolds stress tensor $R^{\left(ref\right)}$:

$$R\left(\alpha_{inlet},u_{*}\right)={\left(\frac{u_{*}^{\left(ref\right)}}{u_{*}}\right)}^{2}\times M\left(\alpha_{inlet}\right)R^{\left(ref\right)}M{\left(\alpha_{inlet}\right)}^{T},$$

$$with\;M\left(\alpha_{inlet}\right)=\left(\begin{array}{ccc} \cos\left(\alpha_{inlet}\right) & -\sin\left(\alpha_{inlet}\right) & 0\\ \sin\left(\alpha_{inlet}\right) & \cos\left(\alpha_{inlet}\right) & 0\\ 0 & 0 & 1 \end{array}\right).$$

#### 4.3.3 Adaptation of the spin-up time

The spin-up time of the LES model has to be adjusted for each sample since the time required to reach a steady state depends on the friction velocity $u_{*}$, since the longer the convective timescale, the longer the time required for the injected eddies to cross the domain. The spin-up time of each simulation $t_{spin-up}\left(\alpha_{inlet},u_{*}\right)$ is therefore set by rescaling the reference spin-up time $t_{spin-up}^{\left(ref\right)}$ by the friction velocity as follows:

$$t_{\mathrm{spin}-\mathrm{up}}\left(\alpha_{\mathrm{inlet}},u_{*}\right)=t_{\mathrm{spin}-\mathrm{up}}^{\left(\mathrm{ref}\right)}\times \left(\frac{u_{*}^{\left(\mathrm{ref}\right)}}{u_{*}}\right).$$

Given the variation range of the friction velocity considered $u_{*}$ (Eq. (2)), the spin-up time of the simulations within the PPMLES dataset simulation varies between 60 and 550 s, as shown in Fig. 2.

### 4.4 Perturbed-parameter ensemble post-processing

In this section, we describe the post-processing performed on the raw LES results, in order to i) estimate their uncertainty, and ii) to reduce the volume of data to store.

#### 4.4.1 Estimation of the uncertainty of the time-averaged fields

Due to the limited analysis period of 200 s, the time-averaged fields predicted by LES are subject to an aleatory uncertainty induced by the internal variability of the atmospheric boundary layer. This irreducible form of uncertainty is significant in the context of the MUST field campaign [4]. To obtain realistic uncertainty estimates for each LES time-averaged field, we use the statistical method designed and validated by [6], which relies on resampling of the sub-averages of the physical fields using the stationary bootstrap algorithm of [13].

We use 1000 bootstrap replicates and the bootstrap block length is set independently for each simulation sample and for each variable to be equal to the spatially averaged correlation time of the variable. For the composite variables $\bar{u^{\prime }c^{\prime }}$, $\bar{v^{\prime }c^{\prime }}$, and $\bar{w^{\prime }c^{\prime }}$, we use the largest correlation time among the correlation times of each variable, in order to avoid uncertainty underestimation. The block length used for each sample and each variable is reported in the Bootstrap_params group of the uncertainty_ave_fields.h5 file. Using this approach, we provide, at each grid node of the domain, an estimate of the aleatory uncertainty associated with each time-averaged field in the PPMLES dataset. This aleatory uncertainty is shown in Fig. 3 for two samples.

#### 4.4.2 Data volume reduction

All the fields in the PPMLES dataset are interpolated onto an analysis mesh with a resolution twice as coarse as the LES mesh. In addition, we restrict the analysis to the circular inner domain D1 (domain with blue boundary in Fig. 8) and below a height of 20 m, since most of the pollutant is located in this area. The corresponding analysis mesh is composed of $1.88\;\times 10^{6}$ nodes, thus reducing the number of nodes by a factor of 10. The characteristic cell sizes of the analysis mesh vary from 0.6 m to 4 m, limiting the loss of information as these resolutions are smaller than the scales of variation of the fields of interest.

To further reduce the volume of data storage volume, we use a scale-offset lossy compression,^6^ which trades precision for storage space by retaining only 16 digits after the decimal point for each floating point in the discretized time-average and uncertainty fields. This reduces the volume of the 3600 time-average and uncertainty field samples from 52.8 Go to 32.1 Go.

These two steps have allowed us to significantly reduce the volume of the dataset, allowing it to be shared and reducing the computational burden associated with its use.

### 4.5 Carbon footprint of the perturbed-parameter ensemble

The computation of the PPMLES dataset was performed on several supercomputers: CERFACS’ Nemo and Kraken, Météo-France's Belenos, and TTGC's Joliot-Curie. The technical characteristics of these supercomputers are summarized in Table 2. The scaling of the LES model was tested for each cluster, resulting in different optimal numbers of cores. In total, the 200 simulations of the perturbed-parameter ensemble have consumed 5.7 milion of core hours.

**Table 2:** Main statistics of the perturbed-parameter ensemble computation and the associated carbon footprint for each supercomputer used. $N_{\mathrm{cpu}}$ is the number of cores on which the LES computations were parallelized, $N_{\mathrm{LES}}$ is the number of LES run on each supercomputer, $Mh_{\mathrm{CPU}}$ is the total computational time in million of core hours, and $\mathrm{tC}O_{2}\mathrm{eq}$ is the associated greenhouse gas emissions in tons of carbon dioxide equivalent.

Supercomputer	Partition	Processors	$N_{CPU}$	$N_{LES}$	$Mh_{CPU}$	$tCO_{2}eq$
Nemo	Haswell	Intel E5–2680v3	600–900	25	0.70	1,4
Kraken	Skylake	Intel 6140	540–900	15	0.34	0,6
Joliot-Curie	Skylake	Intel 8168	1344	49	1.57	2,6
Joliot-Curie	Rome	AMD Epyc7H12	1024	42	1.15	1,9
Belenos	Rome	AMD Epyc7742	1536	69	1.95	3,2

Given the amount of computing resources from high performance computing centers that consume considerable amounts of energy, the PPMLES dataset is thought to be responsible for a substantial amount of greenhouse gas emissions. To raise awareness of this issue, we estimate the carbon footprint of the PPMLES dataset below.

For the simulations performed in CERFACS (20 % of the total), we first estimate the average energy consumption emission factor (i.e. how much greenhouse gas is emitted per core hour of computation). This is obtained by dividing the total greenhouse gas emissions induced by the electricity and cooling consumption of the supercomputers over the year, given the electricity mix of France, by the total number of computing hours performed over the year. In addition, the emissions related to the life cycle of the supercomputers (i.e. manufacturing, transportation and recycling) are known to be of the same order of magnitude, based on two carbon footprint studies: one for a modestly sized supercomputer [14], and one for a partition of a French national computing center (private communication). We therefore estimate the total emission factor of computing in CERFACS to be 2 gCO_2_eq.h_CPU_^-1^ in 2022, from which we derive the greenhouse gas emissions of the LES carried out at CERFACS (Table 2).

For the simulations performed on the TTGC's supercomputers (46 % of the total), we use the GES1point5^7^ carbon footprint estimation tool available for French research laboratories. For the computations performed on Météo-France's supercomputer (34 % of the total), we use the same emission factor as for the Joliot-Curie Rome partition, since they have a similar architecture.

In the end, computing the PPMLES dataset was responsible for the emission of about 9.7 tCO_2_eq, which can be compared to the target of 2 tCO_2_eq/capita to limit global warming to +1.5 °C by 2050. It is worth noting that this estimate is only an order-of-magnitude given the significant uncertainties at involved. It does not include the emissions related to data storage and transfer, which are negligible compared to the computational emissions. The carbon footprint estimate of the PPMLES dataset highlights the substantial environmental impact running large ensemble of high-resolution LES simulations. Strengthening best computing practices is a must to limit this footprint; building community datasets of LES simulations is a further step and allows the pooling of efforts, similar to what has been done for climate simulations [[15], [16]]. The PPMLES dataset is a contribution to encourage the community to move in this direction.

## Limitations

Due to storage limitations, we could not include some fields (e.g. the Reynolds stress tensor components or the concentration maximum) in the PPMLES dataset. However, these fields were stored during the simulations and could be provided by the authors upon request.

More fundamentally, the PPMLES dataset is limited in terms of atmospheric and dispersion conditions compared to what is possible in reality. Only neutral atmospheric conditions have been considered in what can be considered as a first step. It would be interesting to include stable and unstable atmospheric conditions to cover the full range of possible thermal stratification conditions. Furthermore, all LES simulations use the same experimental setup (i.e. the same urban layout and source location). Extending the PPMLES dataset by perturbing more diverse parameters, and thus including a wider range of atmospheric and dispersion conditions, is a direct prospect of this work. It would also be interesting to simulate the same case study using different LES solvers (here only the AVBP LES solver is used). Each LES solver has its own bias, and a variety of LES solvers would introduce structural modeling uncertainties into the dataset, thereby enriching the scientific questions that can be addressed with the dataset.

## Ethics Statement

The authors confirm that they have read and adhere to the ethical requirements for publication in Data in Brief. Additionally, they certify that the current work does not involve human subjects, animal experiments, or any data collected from social media platforms.

## CRediT Author Statement

**Eliott Lumet:** Methodology, Validation, Formal analysis, Visualization, Writing - Original Draft. **Thomas Jaravel:** Methodology, Software, Writing - Review & Editing, Supervision. **Mélanie C. Rochoux:** Conceptualization, Writing - Review & Editing, Supervision.

## Data Availability

ZenodoPPMLES – Perturbed-Parameter ensemble of MUST Large-Eddy Simulations (Original data).

## Footnotes

1 CEDVAL datasets, see https://www.mi.uni-hamburg.de/en/arbeitsgruppen/windkanallabor/data-sets.html (Accessed: 2024-09-23).

2 HDF5 format, see https://www.hdfgroup.org/solutions/hdf5/ (Accessed: 2024-09-19).

3 AVBP LES code, see https://www.cerfacs.fr/avbp7x/ (Accessed 2024-10-09).

4 CENTAUR software, see https://home.centaursoft.com/ (Accessed 2024-10-09).

5 CWIPI software, see https://w3.onera.fr/cwipi (Accessed: 2024-08-19, in French).

6 HDF5 scale-offset lossy compression, see: https://docs.h5py.org/en/stable/high/dataset.html#scale-offset-filter (Accessed: 2024-08-19).

7 GES1point5 tool, see https://apps.labos1point5.org/ges-1point5 (Accessed: 2024-08-19).
